# Violence against children and adolescents by nursing staff: prevalence rates and implications for practice

**DOI:** 10.1186/s13034-020-00350-6

**Published:** 2020-11-06

**Authors:** Ulrike Hoffmann, Vera Clemens, Elisa König, Elmar Brähler, Jörg M. Fegert

**Affiliations:** 1 Department for Child and Adolescent Psychiatry/Psychotherapy, University of Ulm, Steinhövelstr. 5, 89073 Ulm, Germany; 2 Department for Psychosomatic Medicine and Psychotherapy, University Medical Center of Johannes Gutenberg University of Mainz, Maniz, Germany; 3 Department of Medical Psychology and Medical Sociology, University of Leipzig, Leipzig, Germany

**Keywords:** Child maltreatment, Nursing, Medical institutions, Child protection, Child and adolescent psychiatry

## Abstract

**Background:**

International studies show that child maltreatment is a widespread but often underestimated problem that causes high individual, social and economic costs. Child maltreatment is an important topic for the medical sector as well. On the one hand, affected persons often seek support and help from healthcare professionals, but on the other hand, assaults can also occur in medical institutions by healthcare professionals. Surprisingly, there is hardly any data on the frequency of child maltreatment by healthcare professionals in general and particularly by nursing staff.

**Methods:**

Therefore, in a large representative survey of the German population of 2,516 subjects aged between 14 and 91, the experience of child maltreatment in medical institutions by nursing staff was assessed retrospectively.

**Results:**

Of the 46 subjects who had an inpatient stay in a child and adolescent psychiatry before the age of 18, 33.3% reported to have experienced maltreatment by nursing staff, while 17.3% of the 474 persons who had an inpatient stay in general or pediatric hospitals experienced maltreatment by nursing staff. All forms of maltreatment were significantly more frequent in psychiatric compared to general and pediatric hospitals.

**Conclusions:**

The results of our representative retrospective survey demonstrate that maltreatment by nursing staff are not rare individual cases, but that medical facilities bear systemic risks for assault. Therefore, it is necessary that all medical institutions, in particular psychiatric hospitals, address this issue. In order to reduce the risk for assaults, it is important not only to implement structural measures but also to develop an attitude that emphasizes zero tolerance for violence against children and adolescents.

## Introduction

Child maltreatment is defined as “any act or series of acts of commission or omission by a parent or other caregiver that results in harm, potential for harm, or threat of harm to a child” [20] and can be distinguished into 5 subtypes: emotional, physical and sexual abuse and emotional and physical neglect. It can cause severe lifelong health impairment. Victims have an increased risk for cardiovascular and oncologic disorders [7], addiction and mental illnesses [28] as well as social problems such as criminal behavior in adulthood [16]. Often, affected individuals suffer from posttraumatic stress symptoms [24]. Stress and learned helplessness can change brain activity and consequently aggravate or induce PTSD [48]. Reduced self-efficacy [38, 40] and self-blame [25] may lead to increased social, psychological and somatic health problems. Moreover, child maltreatment is associated with altered health behavior encompassing a higher risk for substance abuse and risky sexual behavior [1, 19, 28] and higher rates of smoking [10, 44]. Additionally, biologic mechanisms, such as a dysregulation of the hypothalamic-pituitary-adrenocortical (HPA) axis, a major stress response of the human body [5] and chronic inflammatory processes [2] are discussed to be involved in the pathomechanism mediating childhood maltreatment and later health problems.

The negative consequences for the individual are reflected in economic follow-up costs borne by society: in Germany, the annual maltreatment follow-up costs amount to a margin of 11 to 30 billion Euro [17], in the US to 124 billion US-Dollar [23].

Child maltreatment can occur in different contexts, e.g. in families or—what people might be less aware of—in institutions. Coercive measures in youth residential care were shown to lead to increased aggression, decreased trust and coercive cycles of interaction between group workers and youths [9]. However, there is a lack of data regarding the health sector. In medical institutions, nursing staff engage in various situations with a particular risk for maltreatment such as physical examinations, personal hygiene and other care measures. For the psychiatric sector, the conduction of coercive measures has to be considered additionally. The classification of coercive measures in the context of maltreatment is complex. On the one hand, these can be carried out as medically unnecessary and thus unjustified measures, on the other hand it is possible that they are medically necessary but are perceived as violence by the patients.

Summing up there is a wide range of possible situations in which violence by nursing staff can occur, ranging from not respecting privacy of patients to intentional killings of patients. The latter are presumably rare cases, but are often widely discussed in the public, e.g. [22, 36].

Hardly any study focuses on violence by nursing staff in medical institutions. The few existing studies concerning violence by nursing staff in the medical field mainly deal with the geriatric care sector [49]. Consulting deputy nurse managers in inpatient facilities, 47% of the respondents stated that they consider conflicts, aggression and violence in nursing to be a significant problem in inpatient nursing facilities [11]. In a German study almost one third of the consulted nurses reported measures against the will of patients, residents and those in need of care as common [50].

With a focus on child maltreatment, only few studies assessed prevalence rates of physical violence in different kind of institutions [32, 53–55]. Witt and colleagues estimated prevalence rates of 28% for physical abuse in institutions—16% by caregivers or other personnel [54, 55]. Radford and colleagues estimated rates of 0.1–0.4% for physical violence and rates of 0.3–0.6% for general maltreatment including physical violence, physical neglect and emotional abuse by caregivers in institutions in the U.K. [32]. Witt et al. reported prevalence rates of sexual abuse in schools of 1.4% [52].

It is noticeable that the studies show very different results, which is probably due to the sample and assessment points. For example, in the U.K. survey, only children living in families were assessed, while in the sample of Witt et al. adults were questioned retrospectively. In the latter sample, people who had lived in institutions during their childhood, where prevalence for maltreatment is higher [35], were included in the sample and adults up to an older age participated. Furthermore, in the U.K. survey, parents filled out questionnaires for children under the age of 11, probably thereby missing maltreatment experiences of children [32].

However, both studies did not differentiate between different types of institutions and professionals. In a German population-based analysis, 0.04% of the sample indicated the experience of physical abuse and 0.1% of sexual abuse in hospitals during childhood [54, 55], but this study did not assess whether the perpetrators were hospital staff and in which type of hospitals maltreatment occurred.

Focusing on child and adolescent psychiatry, a German study over a period of 6 years indicated that 9.2% of patients experienced coercive measures. Male patients were slightly in the majority, but female patients who were subjected to coercive measures experienced a significantly higher number of measures [14]. However, different forms of violence were not assessed in this study. Prevalence numbers like these of course don´t say anything about the necessity and adequacy of the coercive measures employed. But as coercive measures constitute a violation of patients’ human rights and are often experienced as such by patients [4, 29, 43], as they can have serious adverse effects ranging from emotional trauma to severe physical injury [3, 4, 26, 51] and as attitudes towards physical violence, both in society in general and in the institutional context, have changed over time [30], efforts are made both in research and clinical practice to identify strategies to reduce coercive measures [8, 15, 18] and to make the measures more understandable for patients, e.g. by patient information. In the field of psychiatric care in Germany, this was legally supported by the Psychiatrie-Enquête ("Report on the Situation of Psychiatry in the Federal Republic of Germany") published in 1975. This was the starting point to improve the situation of people with mental illness in institutional care.

Concerning sexual abuse in institutions, there was an intense debate in media and politics in Germany in 2010. The focus was on educational institutions and facilities of the Catholic Church. As consequences, a politically appointed Round Table committee and the position of an Independent Commissioner were established. In a media campaign, victims of child sexual abuse were requested to report to the Independent Commissioner and to tell their stories. The reports should be used to derive necessary changes in areas such as support and assistance. The accompanying research of the campaign showed that victims had often looked for help from medical professionals such as physicians and therapists. On the other hand, persons also reported that they had experienced sexual assaults by professionals in medical-therapeutic contexts [33]. While the accompanying research provides at least some indications of the frequency and types of sexual assaults by physicians and psychotherapists, there are no corresponding results for assaults by nursing staff. As result of discussions, a number of studies were carried out on the frequency of sexual assaults in institutions. The focus was on educational institutions, the medical sector was hardly considered. This situation of a lack of data has not changed until today. Thus, even though general awareness and research on the topic child maltreatment increases, data regarding maltreatment by staff in the medical field are scarce [31].

In the—to the best of our knowledge—first population-representative study that analyzed the prevalence of child maltreatment by caregivers in several institutions including schools and hospitals, maltreatment specifically by nursing staff in the clinical setting was assessed. 2.2% of the sample indicated to have experienced physical abuse, 2.1% emotional abuse, 0.2% sexual abuse and 2.6% neglect by nursing staff in medical institutions [6]. Using the same population-representative dataset than in the study from Clemens et al. [6], this study takes a detailed look on child maltreatment by nursing staff specifically during inpatient stays in child and adolescent psychiatry. Data are compared to rates in general or pediatric hospitals. As explicated in detail before, this is the first population-representative study analyzing different form of child maltreatment by nursing staff during inpatient stays in different clinical settings.

## Methods and sampling

For this study, the above-mentioned model of classification of the different forms of child abuse defined by the Centers for Disease Control was transferred to the nursing context. According to this adaption, forms of violence in care can be differentiated into maltreatment by nursing staff (institutional abuse) with the three subtypes (1) physical abuse, (2) emotional abuse and (3) sexual abuse and neglect by nursing staff (institutional neglect) with the two subtypes (1) neglect of nursing measures and (2) inadequate supervision. Subsequently, corresponding items were defined for the different subtypes, as no appropriate survey instrument for the specific context of maltreatment by nursing staff was available. The items on physical violence include the assessment of experienced coercive measures.

To assess the prevalence of child maltreatment in institutions, participants had to answer whether they had inpatient stays in a child and adolescent psychiatry or a pediatric or general hospital before the age of 18 years. If any of these questions was affirmed, following questions concerning the experience of child maltreatment were presented. For all items the option “I don’t want to answer this question” was included. The items were assigned to different forms of maltreatment and clustered (See Table 1).

**Table 1:** Forms of maltreatment and assigned items

Items	Form of maltreatment
During this stay in my childhood/youth …	
… I experienced physical violence … •… in form of beating, rough handling during care measures or the like •… in form of coercive measures such as fixation or confinement … by a nurse/nurses (m/f).	Physical abuse
•… I was humiliated, insulted, threatened or intimidated … •… information that I had said in confidence were passed on "behind my back” … by a nurse/nurses (m/f).	Emotional abuse
•… I experienced sexual assaults with penetration •…I experienced sexual assaults without penetration … by a nurse/nurses (m/f).	Sexual abuse
… I had the feeling the nurse/nurses (m/f) … •… was/were not interested in how I was doing. •… did not take sufficient care of me. • … was/were not taking good care of me.	Neglect

Regarding the maltreatment category “neglect”, the item "sufficient care" focuses on the perception of the nurse on the child's or adolescent's needs, whereas the item "good care" focuses on the child´s subjective feeling of safety provided by the nurse.

The items assessing neglect comprise the valid answers “yes”, “no” and “partly”, for the other items only “yes” and “no” were offered as options. For binary analyses, “partly” was taken into the “no” group to have a more conservative estimation of neglect, especially because this was a rather vague wording in this category.

A demographic consulting company (USUMA, Berlin, Germany) obtained a representative sample of the German population using a random route procedure. Data collection took place between May and July 2018. A systematic area sampling was used based on the municipal classification of the Federal Republic of Germany, covering the entire inhabited area of Germany. Households of every third residence in a randomly chosen street were invited to participate in the study. To select participants in multi-person households a Kish-Selection-Grid was applied. For inclusion, participants had to be at least 14 years of age and have sufficient German language skills. The people were informed before inclusion that we were doing a survey on health. Of 4902 initially contacted households, 2516 persons completed the survey (response rate = 51.32%). The main reasons for non-participation were refusal by the selected household to identify the person of target (22.4%, referring to the initial 4902 households), refusal of the target person to participate (12.7%) and failure to contact anyone in the residence after four attempts (13.4%). The resulting sample was representative for the German population above the age of 14 in regard to age and gender.

Individuals who agreed to participate were given information about the study and informed consent was obtained. In the case of minors, participants gave informed assent with informed consent being provided by their caregivers. Participants were told that the study was about psychological health and well-being. Responses were anonymous. In a first step, socio-demographic information was obtained in an interview-format by the research staff face-to-face according to the demographic standards of the Federal Statistical Office. Then, the researcher handed out a copy of the questionnaire and a sealable envelope. This questionnaire was answered independently due to the sometimes very personal information provided. The researcher remained nearby in case the participants needed further information or left the household based on the participants wishes. Anyhow, the researcher did not interfere with filling out the questionnaire. The completed questionnaires were linked to the respondent’s demographic data, but did not contain name, address, or any other identifying information. Socio-demographic questions used for this study were age, gender and inpatient stay in a medical institution before the age of 18 years.

The study was conducted in accordance with the Declaration of Helsinki, and fulfilled the ethical guidelines of the International Code of Marketing and Social Research Practice of the International Chamber of Commerce and of the European Society of Opinion and Marketing Research. The study was approved by the Ethics Committee of the Medical Department of the University of Leipzig.

Of the N = 2516 participants, 1375 (54.5%) were female, 1144 (45.5%) male. Participants were on average 48.0 years old (SD = 17.6, Range 14–91). 85 (3.4%) left school before graduation. 2133 (84.4%) received school graduation as highest academic achievement, 228 (9.1%) an academic degree. 65 (2.6%) of the participants were still attending school. Of the total sample, 46 (4.0%) of the participants reported to have had an inpatient stay in child and adolescent psychiatry and 474 (18.1%) in pediatric or general hospital before the age of 18. These two sub-samples will be in focus for the following analyses.

The characteristics of the sample are presented in Table 2.

**Table 2:** Demographic data of the sample

	Total		Female		Male	
	N		N	%	N	%
	2516		1372	54.5	1144	45.5
Age						
M	48.03		48.52		47.43	
SD	17.57		17.42		17.73	
Range	14–91		14–91		14–89	
**Educational level N (%)**	**N**	**%**	**N**	**%**	**N**	**%**
Left school before graduation	85	3.4	48	3.5	37	3.2
School graduation	2133	84.8	1186	86.4	947	82.8
Academic degree	228	9.1	111	8.1	117	10.2
Attending school	65	2.6	25	1.8	40	3.5
**Inpatient stay before the age of 18…**	**N**	**%**	**N**	**%**	**N**	**%**
Child and adolescent psychiatry	46	1.8	27	2.0	19	1.7
Pediatric or general hospital	474	18.1	278	20.3	196	17.1

All analyses were conducted using SPSS version 21. Descriptive analyses were performed for prevalence rates. For percentages, only valid numbers were included. Total number of the sample is presented for each analysis. Group comparisons for gender were performed via chi^2^-tests or fishers-exact-test depending on the number of observations.

## Results

### Child maltreatment by nursing staff in medical institutions

In psychiatric hospitals, the most frequently reported type of child maltreatment was physical abuse, which was stated by 31.7% of the participants who had been in psychiatric hospitals (f 36%; m 25%). While for males, respective 18.8% reported to have experienced beating, rough handling during care measures or the like as well as coercive measures, for females more reported to have experienced coercive measures (28% versus 16%). For pediatric and general hospital, physical abuse was indicated by 8.6% of the respondents; most of them experienced beating rough handling during care measures or the like (6.5%). As with the psychiatric hospitals, male children were more affected by "beating, rough handling during care measures or the like ", female children more by coercive measures.

Emotional abuse was indicated by 23.1% of the participants who have been in psychiatric hospitals (f 20.8%; m 26.7%), and by 9.5% of the participants who have been in pediatric or general hospital (f 9.9%; m 9.0%). The majority affirmed to have been humiliated, insulted, threatened or intimidated by nursing staff.

Sexual abuse was mentioned by 7.3% of the respondents in child and adolescent psychiatry and 0.7% in pediatric and general hospital. In both types of institutions, male children and adolescents were more frequently affected than female, whereas the difference between the sexes is in psychiatry more distinctive (in psychiatry 12.5% versus 4%, in pediatrics 1.1% versus 0.4%).

22.0% of the respondents who have been in child and adolescent psychiatry and 11.2% of the respondents who have been pediatric and general hospitals reported neglect. Male children and adolescents were more frequently affected than female children (in psychiatry 25% versus 20%, in pediatrics 12% versus 10.7%). The most frequent kind of neglect was the feeling nursing staff did not take sufficient care for both male and female participants.

Comparing the medical institutions, rates for all types for maltreatment were higher in psychiatric hospitals compared to general or pediatric hospitals. The data are presented in Table 3.

**Table 3:** Frequencies of different forms of child abuse differentiated by type of institutions and sex of the victim

	Child and adolescent psychiatry (*N* = 39–41)							Pediatric or general hospitals (*N* = 459–465)						
Types of child maltreatment	Sex of the victim				Chi^2^/Fishers exact	Total		Sex of the victim				Chi^2^/Fishers exact	Total	
	Female		Male					Female		Male				
	n	%	n	%		N	%	n	%	n	%		N	%
**Physical abuse**	9 of 25	36.0	4 of 16	25.0	0.55	13 of 41	31.7	23 of 273	8.4	17 of 192	8.9	0.03	40 of 465	8.6
Beating, rough handling during care measures or the like	4 of 25	16.0	3 of 16	18.8	0.05	7 of 41	17.1	16 of 273	5.9	14 of 192	7.3	0.38	30 of 465	6.5
Coercive measures	7 of 25	28.0	3 of 16	18.8	0.45	10 of 41	24.4	12 of 269	4.5	6 of 192	3.1	0.53	18 of 461	3.9
**Emotional abuse**	5 of 24	20.8	4 of 15	26.7	0.18	9 of 39	23.1	27 of 273	9.9	17 of 189	9.0	0.10	44 of 462	9.5
Humiliated, insulted, threatened or intimidated	5 of 24	20.8	4 of 15	26.7	0.18	9 of 39	23.1	25 of 273	9.2	15 of 189	7.9	0.21	40 of 462	8.7
Passing on information "behind the back" that had been said in confidence	4 of 24	16.7	1 of 16	6.3	0.95	5 of 40	12.5	12 of 268	4.5	9 of 191	4.7	0.01	21 of 459	4.6
**Sexual abuse**	1 of 25	4.0	2 of 16	12.5	1.04	3 of 41	7.3	1 of 271	0.4	2 of 190	1.1	0.81	3 of 461	0.7
Without penetration	1 of 25	4.0	2 of 16	12.5	1.04	3 0f 41	7.3	1 of 271	0.4	2 of 190	1.1	0.81	3 of 461	0.7
With penetration	1 of 23	4.3	2 of 16	12.5	0.88	3 of 39	7.7	1 of 273	0.4	1 of 191	0.5	0.07	2 of 464	0.4
**Neglect**	5 of 25	20.0	4 of 16	25.0	0.50	9 of 41	22.0	29 of 272	10.7	23 of 191	12.0	0.52	52 of 463	11.2
Not interested in how I was doing	4 of 25	16.0	4 of 16	25.0	1.08	8 of 41	19.5	19 of 272	7.0	16 of 191	8.4	3.00	35 of 463	7.6
Did not take sufficient care	3 of 25	12.0	3 of 16	18.8	1.64	6 of 41	14.6	23 of 272	8.5	19 of 191	9.9	2.03	42 of 463	9.1
Did not take good care	3 of 25	12.0	3 of 16	18.8	0.38	6 of 41	14.6	16 of 272	5.9	11 of 191	5.8	1.19	27 of 463	5.8

*N* Number of valid cases

### Changes in attitudes towards violence over time

As described above, the Psychiatrie-Enquête from 1975 has initiated reforms in psychiatric care in Germany, particularly concerning aspects of physical violence and coercive measures. Since the age range of the persons investigated is between 18 and 90 years, it can be assumed that the persons were patients in child and adolescent psychiatry at different historical times. As child and adolescent psychiatry mainly addresses patients until the age of 18 and the Psychiatrie-Enquête came into force 1975, the birth year 1957 (1975 subtracted 18) was used as a marker point. Thus, data of test persons who were born before and including 1957 and consequently had their inpatient stay in psychiatry before the Psychiatrie-Enquête were compared to data of test persons who were born from and including 1958 (inpatient stay in psychiatry after the "Psychiatrie-Enquête" came into force). The results suggest that there was a decrease in physical violence. However, the case numbers are too low to draw clear conclusions about the effects of the legal reforms.

All data are given in Table 4.

**Table 4:** Frequencies of physical abuse in child and adolescent psychiatry differentiated by year of birth before/after 1957^1^

	Child and adolescent psychiatry (*N* = 41)			
	Year of birth up to and including 1957 (N = 7)		Year of birth from and including 1958 (N = 34)	
	n	%	n	%
**Physical abuse**	3 of 7	42.9	10 of 34	29.4
Beating, rough handling during care measures or the like	3 of 7	42.9	4 of 34	11.8
Coercive measures	2 of 7	28.6	8 of 34	23.5

Presented as number of subjects (%). Only valid cases were included. Due to low numbers, no Chi^2^ test was performed

^1^ As child and adolescent psychiatry mainly addresses patients until the age of 18 and the "Psychiatrie-Enquête" came into force 1975, the birth year 1957 (1975 subtracted 18) was used as a marker point for this analysis

### Number of experienced maltreatment forms by nursing staff

The majority of all participants who had inpatient stays in psychiatric hospitals or pediatric/general hospital before the age of 18 experienced no form of maltreatment by nursing staff. As one hypothesis was that coercive measures account for a significant proportion of the reported incidences of maltreatment, the rate was calculated with and without consideration of the item assessing coercive measures. Including coercive measures, 66.7% of the participant who have been in a child and adolescent psychiatry and 82.7% of the participants who have been in a pediatric or general hospital before the age of 18 reported to have experienced no form of maltreatment, 69.2% versus 83.3% excluding coercive measures.

In psychiatric hospitals, 7.7% reported to have experienced one form of child maltreatment, 10.3% to have experienced 2 forms and 7.7% 3 or 4 forms, respectively. In pediatric and general hospitals 8.8% reported to have experienced one form of child maltreatment, 4.8% two forms, 3.3% three forms, and 0.4% to have experienced all four forms of maltreatment.

All data are given in Table 5.

**Table 5:** Number of experienced maltreatment forms by nursing staff

Number of experienced maltreatment forms	Including coercive measures				Excluding coercive measures			
	Child and adolescent psychiatry (N = 39)		Pediatric or general Hospital (N = 456)		Child and adolescent psychiatry (N = 39)		Pediatric or general Hospital (N = 456)	
	n	%	n	%	n	%	n	%
0	26	66.7	377	82.7	27	69.2	380	83.3
1	3	7.7	40	8.8	5	12.8	40	8.8
2	4	10.3	22	4.8	3	7.7	23	5.0
3	3	7.7	15	3.3	1	2.6	11	2.4
4	3	7.7	2	0.4	3	7.7	2	0.4

Presented as number of subjects (%). N = Number of valid cases

### Sex of the perpetrators

While in child and adolescent psychiatry, men are more frequently named as perpetrators for all forms of maltreatment, in pediatric and general hospitals, women are more frequently named as perpetrators for all forms of maltreatment except sexual abuse. For sexual abuse in both types of hospitals only male perpetrators are reported.

The data are presented in Table 6.

**Table 6:** Frequencies of different forms of child abuse differentiated by type of institutions and sex of the perpetrators

Types of child maltreatment	Sex of the perpetrator	Child and adolescent psychiatry		Pediatric or general hospital	
		N	%	N	%
Physical abuse	Male perpetrator(s) involved	9 of 12	75.0	22 of 40	55.0
	Female perpetrator(s) involved	7 of 12	58.3	27 of 40	67.5
Emotional abuse	Male perpetrator(s) involved	6 of 9	66.7	17 of 44	38.6
	Female perpetrator(s) involved	4 of 9	44.4	37 of 44	84.1
Sexual abuse	Male perpetrator(s) involved	3 of 3	100.0	3 of 3	100.0
	Female perpetrator(s) involved	0 of 3	0.0	0 of 3	0.0
Neglect	Male perpetrator(s) involved	7 of 9	77.8	19 of 52	36.5
	Female perpetrator(s) involved	5 of 9	55.6	45 of 52	86.5

Presented as number of subjects (%). Only valid cases were included. The category "female perpetrator involved" includes cases where people indicated the gender of perpetrator(s) as “female” or “female and male”. The category "male perpetrator involved" includes cases where people indicated the gender of perpetrator(s) as “male” or “female and male”

## Discussion

To the best of our knowledge, this is the first population representative study that assesses the prevalence of child maltreatment by nursing staff in child and adolescent psychiatry.

Based on retrospective reports from subjects ages 14–91 years, we have found high prevalence rates of all forms of maltreatment by nursing staff during inpatient stays in child and adolescent psychiatry and pediatric or general hospitals before the age of 18. This is particularly important considering the fact that only a small proportion of the respondents had an inpatient stay in childhood and adolescence at all.

Nearly all frequencies measured in this study are higher than those in the above-mentioned studies. According to our results, the most frequent form of child maltreatment by nursing staff is physical abuse, the rarest sexual abuse. These results are only partly consistent with international studies on child maltreatment, where neglect is consistently identified as the most common form (e.g. US 2004, about 2/3 of the cases; [46]). This inconsistency could be due to the different operationalization of the concept “neglect” in the context of professional nursing compared to the parenting context. In contrast, the operationalization of the other forms of maltreatment are similar for the nursing and parenting context.

Based on our data, there are no significant differences between the proportions of male and female victims, except for sexual abuse. For physical abuse, emotional abuse and neglect our results are consistent with international studies on child maltreatment: The WHO reports in its European Report on Preventing Child Maltreatment that 22.9% of the children are affected by physical abuse and 29.1% by emotional abuse (both sexes equally affected). There are very few studies available for neglect, therefore studies have been included worldwide. Values of 16.3% for physical neglect and 18.4% for emotional neglect were quoted. Again, both sexes are equally affected [39]. For sexual abuse, the WHO study reports more girls than boys as victims, but it comprised sexual abuse in institutions and in family. The fact that the victims of sexual abuse in our sample were more likely to be male matches other reports of sexual abuse in institutions (e.g. Catholic Church), which indicates that boys have a higher risk of becoming victims of sexual abuse in institutions than girls. However, it should be considered that in some of these institutions substantially more or only boys were accommodated [34, 41]. This also applies to the clinical sector in Germany in varying proportions. In child and adolescent psychiatry, boys are clearly overrepresented in the age group up to 10 years with 73.4%, in the older age groups this shifts to a distribution of approx. 45% boys and 55% girls. In pediatric or general hospitals this constellation (boys approx. 55%, girls approx. 45%) applies to all age groups [42]. Moreover, the number of participants in our study who reported to have experienced sexual abuse was low, so the validity of our data is limited and more research is needed in this field.

The rates for all forms of maltreatment and also the risk of being affected by more than one form of child maltreatment by nursing staff were considerably higher in the psychiatric field than in somatic hospitals. There are various possible explanations for this. Often these patients lack support and care from parents or other caregivers and have only few external contacts, so that they find themselves in a particularly vulnerable situation and have limited opportunities to complain. Another reason could be, that patients in child and adolescent psychiatry are stigmatized because of their mental disorder and the associated symptoms.

Furthermore, relationships between nursing staff and patient in child and adolescent psychiatry are characterized by a deeper intensity and intimacy [27, 45], which can increase the risk of maltreatment. The emotional dependence on the nursing staff is higher than in pediatric and general hospitals, as they are involved, for example, in decisions about participation in social activities or the use of mobile phones. Conceptually, higher emotional dependence could also be a protective factor, but research shows that higher emotional dependence rather results in a higher risk of e.g. sexual abuse as it facilitates typical grooming strategies of the perpetrator [21].

In Germany, the average length of stay in pediatric hospital is 4.5 days, in child and adolescent psychiatry 34.4 days, so almost 7.6 times higher [42]. As the duration of inpatient stays is considerably longer in psychiatry compared to somatic hospitals, there are more opportunities for assaults in psychiatric hospitals due to the very fact that the patients spent a greater number of days there. In contrast, one could argue, that clinics with longer inpatient stays hold better chances to implement safeguarding measures protecting patients from maltreatment. Although it is true, that lower fluctuation of patients facilitates implementation of safeguarding measures, it also increases potential risky situations and dependency of patients.

The results show that a significant proportion of respondents who had an inpatient stay in child and adolescent psychiatry experienced coercive measures. With 24.4%, the rate was considerably higher than indicated in the study by Fetzer et al. [14]. It is conceivable that medically necessary coercive measures were experienced as violence by the children and adolescents because they were not sufficiently informed about the necessity of the measure or were not able to understand the current medical need due to their disorder. For clinical practice, this means that patients should always be informed about the necessity of coercive measures and the measures should be discussed after completion. The aim should be to avoid them if at all possible.

As consequence of this finding, that there was a high prevalence of experienced coercive measures, we had the hypothesis that a large proportion of the frequencies of physical abuse was due to the exposure to coercive measures. In child and adolescent psychiatry, 33.3% have experienced at least one form of maltreatment, while in pediatric and general hospitals the figure was 17.1%, without consideration of coercive measures the figures are 30.8 and 16.4% respectively. Thus, the hypothesis that a high proportion of physical abuse is due to coercive measures was not confirmed. However, recent public debates in Germany about the use of coercive measures show that child and adolescent psychiatric clinics differ significantly in their application practice of coercive measures and that there is no standardized approach with regard to the frequency and clinical indications of the use of coercive measures [12].

Our results suggest, that application of coercive measures and physical abuse in general decreased in the course of reforms in psychiatric care initiated by the “Psychiatrie-Enquête”, but due to low number of cases, no clear conclusions could be drawn. However, this interpretation is underlined by a previous analysis that included subjects in all assessed kind of hospitals without sub stratification for psychiatry, showing that oldest participants reported highest rates of maltreatment [6].

The evaluation of data on the sex of the perpetrators showed heterogeneous results and should be interpreted with caution, because of the rather small sample size. While in child and adolescent psychiatry the perpetrators were mostly male, apart from neglect, in pediatric and general hospital, they are mostly female, apart from sexual abuse. To contextualize these data, it is necessary to consider the gender distribution of professionals in the two areas somatic hospitals and child and adolescent psychiatry. In Germany, the ratio of female to male nurses specialized in child healthcare is 97/3% in somatic hospitals and 87/13% in child and adolescent psychiatry [42]. Considering these figures, in our sample male perpetrators committed all types of child maltreatment with above-average frequency.

One reason for the increased frequency of male perpetrators in physical abuse could be the application of coercive measures, where male nurses are more often involved due to their physical strength. For the context of violence against children in institutions by nursing staff, perpetrators may specifically select these occupational fields in order to be able to exercise power against children and adolescents.

In the reported cases of sexual violence, only men were mentioned as perpetrators. A higher rate had been expected here already based on international studies, showing that sexual abuse is committed by men and male adolescents in about 80 to 90% of cases [47]. Sexual abuse by women has only recently come into the focus of research. However, the question is whether sexual assaults by women (or rather activities that are carried out with sexual intent) are recognized as such by children and adolescents at all.

### Limitations

One of the limitations of the study is to define suitable items, especially in the area of neglect. This may lead to confusion by responders and potential unintended alternative interpretation of the items and decrease the validity of our results.

Another limitation it that the results are based on a retrospective self-report. In all retrospective analyses, there is a potential for underreporting due to potential distortion of memory content and recall bias. This can be the result of denial due to e.g. symptoms of posttraumatic stress disorder and self silencing, embarrassment and misunderstanding and can lead to an underestimation of the presented results [13]. Furthermore, it can be assumed that the number of unreported cases of violence by nursing staff is higher than the results suggest. Reasons for this can be related to limited memory capacities, for example, respondents who were in hospitals at a young age usually do not remember their stays, there may have been situations of limited consciousness in which assaults occurred etc.

Another factor may be that patients usually cannot assess which nursing measures are necessary and useful and whether they have been carried out with accuracy. This can result in both sexual assaults e.g. in the form of unnecessary physical examinations and care measures and neglect of care measures. Another factor that may contribute to underestimating prevalence is that some of the respondents are not yet 18 years old and may still experience maltreatment up to the age of 18.

A major weakness of the study is that the findings are based on a rather low sample size so that validity can be impaired. Another limiting factor for the validity is our response rate of 50%. Nevertheless, our results give an important first insight into maltreatment by nursing staff in institutions.

### Implications for practice

The high prevalence rates assessed retrospectively in a population representative sample above the age of 14 show that violence by nursing staff are not rare individual cases, but that hospitals and even more significantly psychiatric hospitals feature inherent risks for child maltreatment. Therefore, it is important that hospitals acknowledge and address this topic by implementing preventive measures.

In Germany, following the abuse scandal in 2010, there was an intensive debate about protection against sexual violence in institutions. An appointed Round Table Committee demanded that structural measures must be implemented in institutions to protect children and young people from (sexual) abuse. These measures are summarized in German by the term "Schutzkonzepte", in English “safeguarding measures”. The Round Table Committee defined in its final report components for such concepts, which have to be further developed and adapted specifically by each institution to its own context [37]. Examples for such measures are a mission statement, a code of conduct or a comprehensive complaint system. At first, it is necessary to carry out a risk analysis to identify situations at high risk for the different forms of child maltreatment and then to establish measures to minimize the identified risks. Each institution must conduct this analysis specifically, because risk factors are different in various medical institutions. For example, the prevalence of coercive measures in psychiatry is much higher than in departments for somatic disorders.

The data collected here show that in the context of such organizational developments not only sexual abuse should be considered, but also all other forms of child maltreatment. In addition, it is necessary to establish zero tolerance approach for violence against children and adolescents. Likewise, there is a need for more research with larger sample sizes and specializing on forms of violence in different types of hospitals.

## Data Availability

The datasets generated and analysed during the current study are not publicly available but are available from the corresponding author on reasonable request. **Ethics approval and consent to participate** The study was approved by the Ethics Committee of the Medical Department of the University of Leipzig. Informed consent was assured.
